# Karyotype and sex chromosome differentiation in two *Nalassus* species (Coleoptera, Tenebrionidae)

**DOI:** 10.3897/CompCytogen.v10i3.9504

**Published:** 2016-09-02

**Authors:** Dirim Şendoğan, Nurşen Alpagut-Keskin

**Affiliations:** 1 Ege University, Faculty of Science, Department of Zoology, Section of Biology, Bornova, Izmir 35100 TURKEY

**Keywords:** Karyotype, Tenebrionidae, Helopini, Nalassus bozdagus, Nalassus plebejus, sex chromosomes, heterochromatin, NOR, DAPI

## Abstract

Cytogenetic features of *Nalassus bozdagus* Nabozhenko & Keskin, 2010 and *Nalassus plebejus* Küster, 1850 were analysed using conventional and differential staining. Mitotic and meiotic chromosomal analysis revealed the diploid number as 2n = 20 (9+Xy_p_) in both species. Besides the general resemblance of two *Nalassus* Mulsant, 1854 karyotypes, important differences related to variations in the number of metacentric/submetacentric chromosomes, localization of highly impregnated regions which are considered as NOR and heterochromatin distribution are clearly observed. The most prominent difference between two species is found related to the X chromosome which is clearly larger in *Nalassus bozdagus* and has a conspicuous secondary constriction on the long arm. As a result of silver staining, the existence of highly impregnated areas associated with Xy_p_ of *Nalassus bozdagus* in both prophase I and metaphase I, suggests that NORs are seemingly located on sex chromosomes. On the other hand, the potential NORs of *Nalassus plebejus* were observed only in prophase I nuclei. With the application of fluorescence dye DAPI, the AT rich chromosome regions and Xy_p_ which forms the parachute configuration were shown in both species.

## Introduction

In the light of fossil and molecular dating analysis, the darkling beetles are dispersed and diversified over the last 180 million years prior to Gondwanan fragmentation. Tenebrionids represent a hyperdiverse family of Coleoptera with ca. 20000 recognized species worldwide. In consequence of undergoing multiple evolutionary radiations, tenebrionids show considerable morphological variations and several adaptations in life history traits such as feeding behaviour, habitat preferences, flight ability etc. Although higher level of tenebrionid phylogeny based on sequences from seven out of nine subfamilies shows well supported monophyly, the subfamilies Diaperinae, Pimeliinae and Tenebrioninae were recovered as paraphyletic or polyphyletic (Kergoat et al. 2014a, b).

The karyotypes of more than 250 darkling beetle species have been determined (Holecová et al. 2008a, Juan and Petitpierre 1991a, Blackmon and Jeffery 2015, Gregory 2016). Although most species present a karyotype with 2n = 20, the diploid number ranges from 2n = 14 to 2n = 38 (Juan and Petitpierre 1991a, Pons 2004, Holecová et al. 2008a, Lira-Neto et al. 2012). Chromosomal data are only available for several representatives of subfamilies Lagriinae, Tenebrioninae, Pimeliinae, Alleculinae and Diaperinae mostly distributed in Mediterranean (Juan and Petitpierre 1991a).

The genus *Nalassus* Mulsant, 1854 (Tenebrioninae: Helopini) comprises 71 described taxa distributed mainly in the Western Palearctic, but with disjunctively isolated species in the Russian Far East and Northern China (Medvedev 1987, Nabozhenko 2001). Even though a significant part of the species is found in alpine and subalpine mountainous belts with high level of local endemism, some species that are adapted to lowlands have wider distribution. In the recent reviews of *Nalassus* species from the European part of CIS (Commonwealth of Independent States), Caucasus, Iran, Georgia, China and Turkey, several new species and combinations were also noted (Nabozhenko 2001, Nabozhenko 2007, Nabozhenko 2008, Keskin and Nabozhenko 2010, Nabozhenko 2011, Nabozhenko 2013, Nabozhenko 2014, Nabozhenko and Ivanov 2015). Therefore, the actual diversity is certainly higher than previously estimated and the monophyly of the genus *Nalassus* needs to be tested with several new additional characters. The chromosomes of *Nalassus* have not yet been studied. Furthermore, cytogenetic data concerning the tribe Helopini which provide no more than chromosome numbers and sex determination systems are only known for some *Nesotes* and *Probaticus* species (Juan and Petitpierre 1986, 1989, 1991a, 1991b, Palmer and Petitpierre 1997).

In this study, with the aim of providing first cytogenetic information about *Nalassus*, the mitotic and meiotic chromosomes of endemic *Nalassus bozdagus* and widespread *Nalassus plebejus* were analysed using conventional, DAPI fluorochrome staining and silver impregnation.

## Material and methods

The meiotic and mitotic chromosomes of 12 male *Nalassus plebejus* and 4 male *Nalassus bozdagus* individuals from Western Anatolia were analysed. The specimens of *Nalassus plebejus* were retrieved from Ballıkayalar Natural Park, Gebze (40°50'22.96"N / 29°30'56.11"E, 120m) and the specimens of *Nalassus bozdagus* were collected from Bozdağ, İzmir (38°15'17.49"N / 27°57'44.72"E, 2300m). Adult beetles were collected on the trunks of trees and on the ground at night when they are active.

The chromosome preparations were obtained from the gonads of male specimens using Murakami and Imai’s (1974) splashing method with some modifications. Briefly, testes were carefully dissected and macerated with sterilized needles. Testes were treated with hypotonic solution (0.65% KCl) for 5 minutes and fixed in 3:1 ethanol: acetic acid at least for 1 h on ice.

We also applied a microspreading method (Chandley 1994) for obtaining prophase I nuclei. The slides were stained with 4% Giemsa in phosphate buffer pH 6.8, for 20 minutes for standard staining. The silver impregnation technique of Patkin and Sorokin (1984) was performed to determine the possible NOR regions. Briefly, slides were incubated in distilled water for 30 min. at room temperature and stained with AgNO_3_ working solution (2:1, 50% AgNO_3_: 2% gelatin containing 0.5% formic acid) in a humid chamber at 60 °C for 3-10 minutes. After a golden-brown color has developed, the reaction was stopped by rinsing with distilled water. Slides were then dehydrated, counterstained with 4% Giemsa in phosphate buffer pH 6.8.

For determining of heterochromatin distribution, the slides were mounted with antifade mounting medium with fluorochrome DAPI (Vectashield) specific to AT-rich chromosomal regions. The visualization of DAPI stained plates were carried out with Olympus BX50 fluorescent microscope.

The mitotic and meiotic plates were analysed and photographed with Zeiss Axio Scope light microscope using ZEN software. The chromosomal measurements were made with the LEVAN plugin (Sakamoto and Zacaro 2009) and the karyotypes and idiograms were created with the CHIAS plugin (Kato et al. 2011) of the programme IMAGE J (Rasband 1997-2015).

## Results

### Conventional Giemsa staining

**Figure 1.**
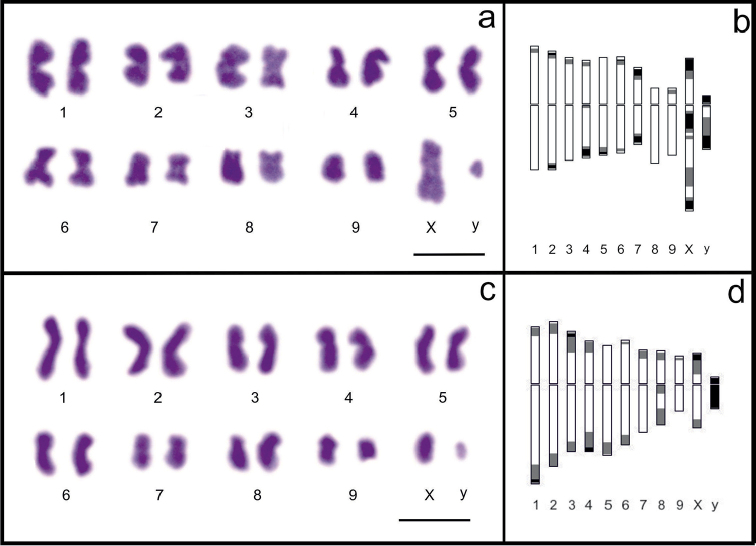
**a–b** *Nalassus bozdagus* 2n = 20. **a** karyotype **b** idiogram **c–d** *Nalassus plebejus* 2n = 20 **c** karyotype **d** idiogram. Bar = 5 µm.

Analysis of spermatogonial cells of *Nalassus bozdagus* and *Nalassus plebejus* revealed the diploid chromosome number as 2n = 20 (9+Xy_p_) (Fig. 1). In both species, most of the autosomes showed metacentric morphology, the X chromosomes were submetacentric and the y chromosomes were subtelocentric. In *Nalassus bozdagus* the autosomal pairs 8 and 9 were submetacentric while in *Nalassus plebejus* autosomal pairs 1, 5 and 8 were submetacentric. The smallest chromosome in both species was determined to be the y chromosome (~1 µm). The biggest chromosome of *Nalassus bozdagus* was the X chromosome (~4.315 µm), in *Nalassus plebejus* the biggest chromosome was the 1. chromosome with the length of 4.442 µm (Table 1).

**Table 1.** Chromosome morphologies and measurements of *Nalassus bozdagus* and *Nalassus plebejus*. CI: centromere index, RL: relative length, AR: arm ratio, *secondary constriction.

	*Nalassus bozdagus*					*Nalassus plebejus*				
Chromosome	Length (µ)	CI	%RL	AR	Morphology	Length (µ)	CI	%RL	AR	Morphology
1	3.895	46	12.4	1.20	m	4.442	35	14.26	1.8	sm
2	3.420	47	10.89	1.23	m	4.207	48	13.5	1.04	m
3	3.375	46	10.74	1.29	m	3.316	43	10.64	1.4	m
4	3.204	46	10.2	1.10	m	3.117	43	10	1.28	m
5	2.876	47	9.16	1.20	m	3.222	28	10.34	2.52	sm
6	2.715	48	8.64	1.06	m	3.040	44	9.76	1.27	m
7	2.204	48	7.02	1.07	m	2.439	45	7.83	1.18	m
8	2.162	33	6.88	2.13	sm	2.476	29	7.95	2.38	sm
9	2.149	32	6.84	2.01	sm	1.853	46	5.95	1.17	m
X	4.315	28	13.74	2.47	sm*	2.04	30	6.55	2.31	sm
y	1.097	20	3.5	3.88	st	1.010	18	3.24	1.17	st

**Figure 2.**
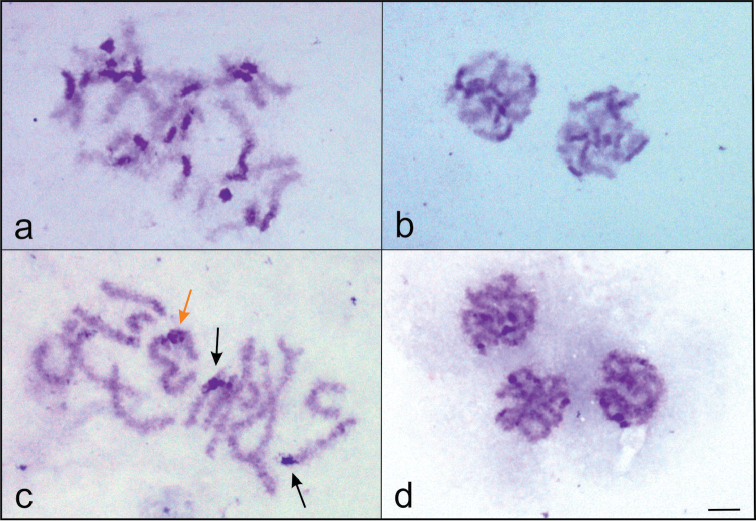
**a–b** *Nalassus bozdagus* with dark heterochromatic blocks on all chromosomes. **a** leptotene–zygotene **b** pachytene **c–d** *Nalassus plebejus* with two distinctive heterochromatic blocks (black arrows); **c** leptotene–zygotene **d** pachytene. Orange arrow indicates Xy_p_ sex bivalent, Bar = 5 µm.

In prophase I nuclei, all chromosomes of *Nalassus bozdagus* showed dark heterochromatic blocks mainly located in centromeric regions (Fig. 2a–b). But in *Nalassus plebejus*, while most of the chromosomes have relatively small amounts of heterochromatin dispersed throughout the whole length (Fig. 2c–d), only 2 chromosomes with distinctive heterochromatic blocks were observed.

**Figure 3.**
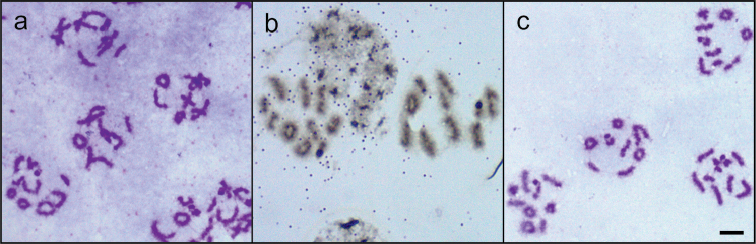
**a** diplotene–diakinesis in *Nalassus plebejus* **b–c** diakinesis–metaphase I **b** *Nalassus bozdagus* **c** *Nalassus plebejus*. Bar = 5 µm.

**Figure 4.**
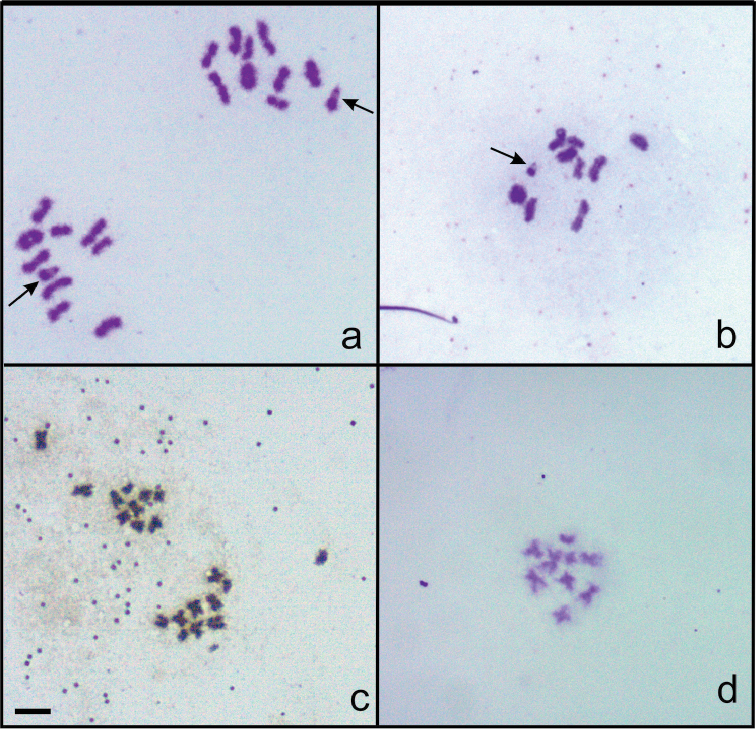
**a–b** metaphase I **a** *Nalassus bozdagus* **b** *Nalassus plebejus* **c–d** metaphase II **c** *Nalassus bozdagus* **d** *Nalassus plebejus*. Arrows show Xy_p_ sex bivalents, Bar = 5 µm.

In diplotene/diakinesis of *Nalassus plebejus*, 5-6 rod-shaped (terminal chiasma), 2-3 ring-shaped (two terminal chiasmata) and 1-2 cross-shaped (interstitial chiasma) bivalents were observed (Fig. 3a). In diakinesis/metaphase I; most of the homologous chromosomes of both species formed rod shaped bivalents due to being monochiasmatic and 2-3 ring shaped bivalents due to being bichiasmatic (Fig. 3b–c). In metaphase I plates, the parachute formation of sex bivalents was clearly observed for both *Nalassus* species (Fig. 4a–b). In metaphase II plates, relatively small sized 10 chromosomes (Fig. 4c–d) were observed. However, the plates which possessed the minute y chromosome were seemed to have only 9 chromosomes in their haploid sets. (Fig. 4d).

Sex chromosomes of two species were differed from each other by the length of X chromosome. The X chromosome of *Nalassus bozdagus* was determined to be almost twice the size of the X chromosome of *Nalassus plebejus* (Fig. 1, Table 1).

### Differential staining

**Figure 5.**
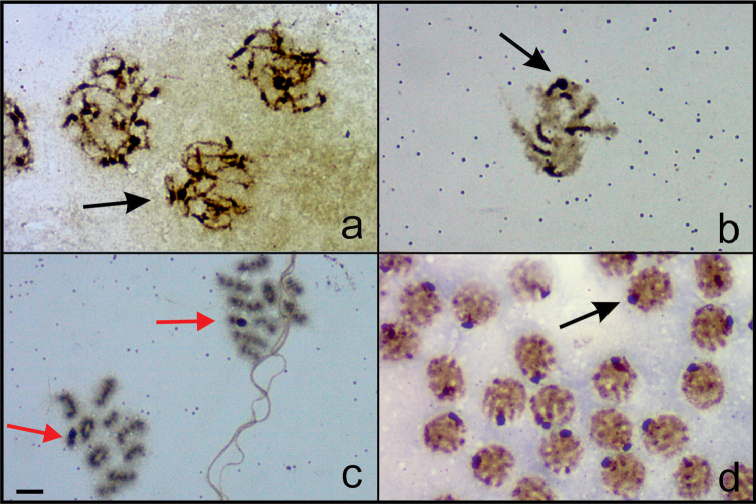
Silver nitrate staining **a–c** *Nalassus bozdagus* **d** *Nalassus plebejus*. Black arrows indicate NOR, red arrows indicate argyrophilic sex bivalents, Bar = 5 µm.

**Figure 6.**
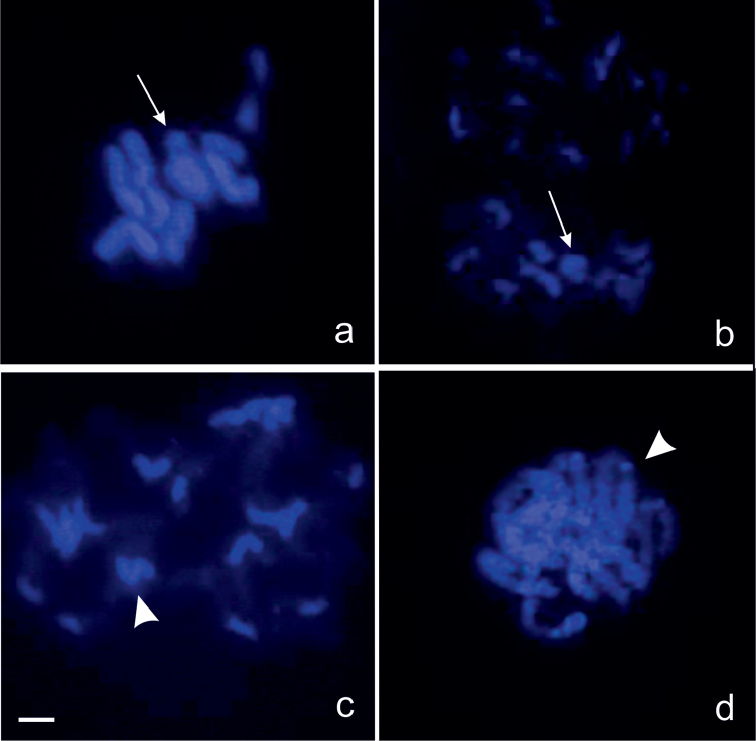
DAPI staining **a** metaphase I of *Nalassus plebejus* **b** metaphase I of *Nalassus bozdagus* **c** prophase I nucleus of *Nalassus bozdagus* **d** prophase I nucleus of *Nalassus plebejus*. Arrows show Xy_p_ sex bivalents and arrowheads indicate heterochromatic regions, Bars = 5 µm.

Silver nitrate staining of the chromosomes of *Nalassus bozdagus* revealed the presence of a highly impregnated nucleolus (NOR) associated with one of the long chromosomes in prophase I nuclei (Fig. 5a–b) and that Xy_p_ sex bivalent is strongly argyrophilic in metaphase I (Fig. 5c). In *Nalassus plebejus*, these strongly argyrophilic regions were observed only in pachytene nuclei (Fig. 5d). With base-specific (A-T) DAPI staining; metaphase I plates and prophase I nuclei were observed. In metaphase I stages there were no significant difference between species (Fig. 6a–b). Prophase I nuclei of *Nalassus bozdagus* showed strong signals on pericentromeric heterochromatic blocks compared to other chromosomal regions (Fig. 6c). On the other hand, in *Nalassus plebejus* only some centromeric regions showed slightly stronger fluorescence signals (Fig. 6d).

## Discussion

The family Tenebrionidae is considered a karyologically conservative group due to the frequent occurrence of 2n = 20 formula (Juan and Petitpierre 1991a, Palmer and Petitpierre 1997). Heretofore, variation in the diploid chromosome numbers between 14–38 within the family are also noted (Juan and Petitpierre 1991a, Pons 2004, Holecová et al. 2008a, Lira-Neto et al. 2012). Although, tenebrionid karyotypes characterized with predominant presence of metacentric chromosomes (Guenin 1950, 1951a, b; Smith 1952, Yadav and Pillai 1974, Yadav et al. 1980, Juan and Petitpierre 1988, 1989, 1990, Juan et al. 1989), several species from different subfamilies have mostly subtelocentric/acrocentric sets (e.g. *Laena reiteri* Weise 1877, 2n = 18, Holecová et al. 2008a, *Palembus dermestoides* Fairmaire 1893, 2n = 20, Almeida et al. 2000). Furthermore, many tenebrionid beetles possess similar chromosome number but differ in karyotype structure, which reveal additional evidence for karyotype divergence through the intra-chromosomal rearrangements. The major patterns of karyological variations in tenebrionid beetles are mainly observed in sex determining systems, chromosome morphology and distribution of heterochromatin (Juan and Petitpierre 1990, Petitpierre et al. 1991, Juan and Petitpierre 1991a-b, Juan et al. 1993, Bruvo-Madaric et al. 2007).

Tenebrionidae comprises 9 subfamilies but most of the cytogenetically studied species (96%) belong to the Pimeliinae and Tenebrioninae (Bouchard et al. 2005, Holecová et al. 2008a). The diploid number in Pimeliinae shows a decrease from 2n = 20 to 2n = 18 caused by fusion of an autosomal pair while in Tenebrioninae there is a tendency of increased diploid number probably caused by centric fissions (Juan and Petitpierre 1991a).

We showed here that the karyotypes of *Nalassus bozdagus* and *Nalassus plebejus* consist of 10 pairs of chromosomes (2n = 20) (Fig. 1), which is considered as modal chromosome number for Tenebrionidae (Juan and Petitpierre 1991a, Pons 2004, Holecová et al. 2008a, Lira-Neto et al. 2012). The presence of heteromorphic sex chromosomes for both species is confirmed by occurrence of a Xy_p_ configuration in both conventionally (Fig. 4a-b) and differentially (Fig. 5c, 6a–b) stained metaphase I plates. The Xy_p_ sex determining system is the most frequent type among Tenebrionidae as well as order Coleoptera (Smith and Virkki 1978, Juan and Petitpierre 1991a). However, sex chromosomes or sex determining systems are mentioned as one of the major chromosomal changes involved in tenebrionid divergence.

Besides the general resemblance of two *Nalassus* karyotypes, important differences related to X chromosomes, variations in the number of metacentric/submetacentric chromosomes (Fig. 1, Table 1), localization of highly impregnated regions which are considered as NOR (Fig. 5a–d) and heterochromatin distribution (Fig. 6c-d) are clearly observed. The most prominent difference between two species is found related to X chromosome which is clearly larger (13.74% of total complement) in *Nalassus bozdagus* and has a conspicuous secondary constriction on the long arm (Fig. 1). It was also observed that metaphase I plates of *Nalassus bozdagus* have relatively larger Xy_p_ (Fig. 4a–b). The increase in relative length of X which does not alter parachute configuration is named as giant Xy_p_ and generally thought to be derived from either heterochromatin amplification or translocation (Dutrillaux and Dutrillaux 2009). Difference in size and heterochromatin content of X chromosomes also observed in two closely related tenebrionid species of *Gonocephalum* Solier 1834 (Tenebrioninae) (Juan and Petitpierre 1989).

The differences found in chromosome morphology (1., 5. and 9. pairs) between these two *Nalassus* species are thought to be related to pericentromeric inversions that resulted in centromeric shift. Pericentromeric rearrangements are already known within several Coleopteran families such as Cicindelidae, Chrysomelidae, Meloidae, Scarabaeidae and Tenebrionidae (Serrano 1981, Petitpierre 1983, Juan et al. 1990, Almeida et al. 2000, Petitpierre and Garneria 2003, Wilson and Angus 2005, De Julio et al 2010, Petitpierre 2011).

The karyotypes of *Nalassus bozdagus* and *Nalassus plebejus* also show obvious differences, especially in distribution of heterochromatin. The presence of strong signals on pericentromeric heterochromatin blocks on all chromosomes of *Nalassus bozdagus* (Fig. 2a–b) and only few chromosomes in *Nalassus plebejus* (Fig. 2c–d) was supported with both conventionally and differentially stained prophase I nuclei (Fig. 4g–h). Although, occurrence of heterochromatin observed mainly in the pericentromeric areas of the tenebrionid chromosomes, variability of heterochromatin localization and composition were also reported (Juan and Petitpierre 1989, 1991, Pons 2004, Rozek et al. 2004, Cabral-de-Mello et al. 2010, Schneider et al. 2007).

As a result of silver staining, the existence of highly impregnated areas associated with Xy_p_ of *Nalassus bozdagus* in both prophase I and metaphase I, suggests that NORs are seemingly located on sex chromosomes (Fig. 5a–c). On the other hand, the potential NORs of *Nalassus plebejus* were observed only in prophase I nuclei (Fig. 5d). Similar findings on argyrophilic Xy_p_ in metaphase I as well as prophase I were previously reported for several beetles such as Zophobas aff. confusus Gebien 1906 (Tenebrionidae) (Lira- Neto et al. 2012), *Lagria villosa* Fabricius 1781 (Tenebrionidae) (Goll et al. 2013), *Palembus dermestoides* (Tenebrionidae) and *Epicauta atomaria* Germar 1821 (Meloidae) (Almeida et al. 2000). Although, rDNA-FISH studies has shown that these strongly argyrophilic areas in prophase I bivalents are associated with NOR (Juan et al. 1993, Bruvo-Madaric et al. 2007), the existence of highly impregnated areas on sex chromosomes until metaphase I thought to be related to association and segregation of sex bivalents due to nucleolar material or distinctive heterochromatin (Juan et al. 1993, John and Lewis 1960). The association between sex chromosomes and nucleolar material is widely known for several animal groups from mammalians to insects (Smith and Virkki 1978, Virkki et al. 1991, Tres 2005), although autosomal localization of NORs by FISH using 18S rDNA probes were also reported for some tenebrionid species (Goll et al. 2003, Juan et al. 1993).

It was observed that bichiasmatic autosomes form ring bivalent while monochiasmatics form rod bivalents due to terminal chiasmata (Fig. 3b–c). Ring bivalents are frequent among Tenebrionidae, Scarabaeidae, Meloidae, Buprestidae, Curculionidae, Chrysomelidae and Cerambycidae (Petitpierre 1985, Bisoi and Patnaik 1988, Petitpierre and Garneria 2003, Karagyan et al. 2004, 2012, Lachowska et al. 2004, 2006a, 2006b, Rozek et al. 2004, Angus et al. 2007, Holecová et al. 2008b). During diploten-diakinesis of *Nalassus plebejus*, in addition to ring and rod bivalents we also observed cross shaped bivalents (Fig. 3a) due to interstitial chiasmata.

The information acquired from metaphase I plates of only few *Nesotes* Allard 1876 species (Juan and Petitpierre 1986, 1989, 1991a, 1991b) and *Probaticus ebeninus* A. Villa and J. B. Villa, 1838 (Palmer and Petitpierre 1997) are the only cytogenetic data concerning the tribe Helopini. On the basis of metaphase I plates, it was only briefly noted that 5 *Nesotes* species have similar 2n = 20, 9+ Xy_p_ formula (Juan and Petitpierre 1986, 1989, 1991a) and *Probaticus ebeninus* have 2n = 20, 9+XY (Palmer and Petitpierre 1997). Although, our findings for chromosome numbers correspond to previous cytogenetic data, comparative genomic analyses of Helopini require detailed descriptions of chromosome morphologies.

In conclusion, this study revealed that the cytogenetic features differed between endemic *Nalassus bozdagus* and widespread *Nalassus plebejus*. But, in the absence of molecular cytogenetic and phylogenetic approaches, it is not possible to make a strong conclusion about the major forces underlying these chromosomal variations. For definitive testing of the general trends in both *Nalassus* and tenebrionid karyotype evolution, it is necessary to increase the taxa sampling for major tenebrionid lineages.
